# Delayed Presentation of Complete Ileal Transection Following Blunt Trauma Abdomen: A Condition to Cognize

**DOI:** 10.7759/cureus.5973

**Published:** 2019-10-23

**Authors:** Sudharsanan Sundaramurthi, Shankar H, Nagarajan Raj Kumar, Shanmugam Dasarathan, Kadambari D

**Affiliations:** 1 Surgery, Jawaharlal Institute of Postgraduate Medical Education and Research, Puducherry, IND; 2 Surgery, Jawaharlal Institute of Postgraduate Medical Education and Research , Puducherry, IND

**Keywords:** ileal perforation, blunt trauma abdomen, crush injury, mesenteric injury, ischemic necrosis, small intestine

## Abstract

Isolated small bowel perforation is a rare presentation of blunt abdominal trauma, and most cases present immediately following the trauma. Delayed presentation of such cases beyond one week of trauma is extremely rare, and various pathophysiological mechanisms were described for the same. We present a 20-year-old male patient who sustained blunt abdominal and pelvic trauma, underwent open reduction and internal fixation for right acetabular fracture, and later developed features of acute peritonitis after one month. On laparotomy, complete terminal ileal transection was found and an ileostomy was done. Delayed perforation of the intestine following trauma occurs due to ischemic necrosis, either through direct trauma to the intestinal wall or indirectly by injury to the mesenteric vessels. Direct trauma to the bowel can result in large hematomas on the bowel wall, which can later perforate due to ischemia. Surgeons should be aware of this rare presentation as the management is challenging and it poses significant medico-legal sequel. Close monitoring of the patient’s vitals and examination for the development of abdominal signs along with repeat imaging at the onset of abdominal signs are cornerstones for successful management of these patients.

## Introduction

Blunt abdominal trauma is a challenging condition causing significant morbidity and mortality, especially in developing countries. The delayed onset of symptoms and signs in these patients contributes greatly to the increased morbidity and mortality in these patients. We present a rare case of delayed presentation of small bowel injury following blunt abdominal trauma and has outlined the pathogenesis and management of such presentations.

## Case presentation

**Figure 1:**
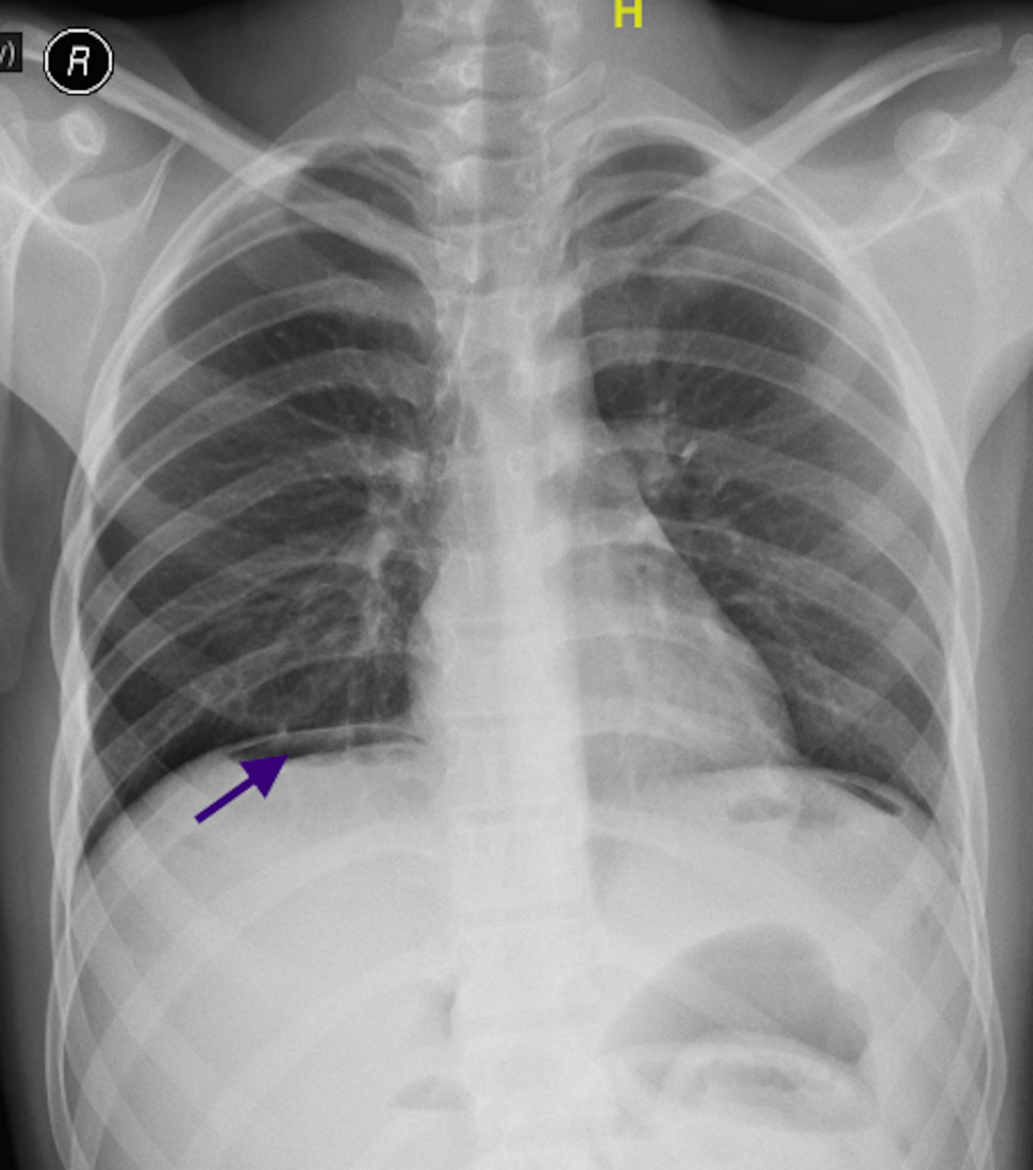
Chest X-ray erect view shows air under diaphragm (marked by arrow)

**Figure 2:**
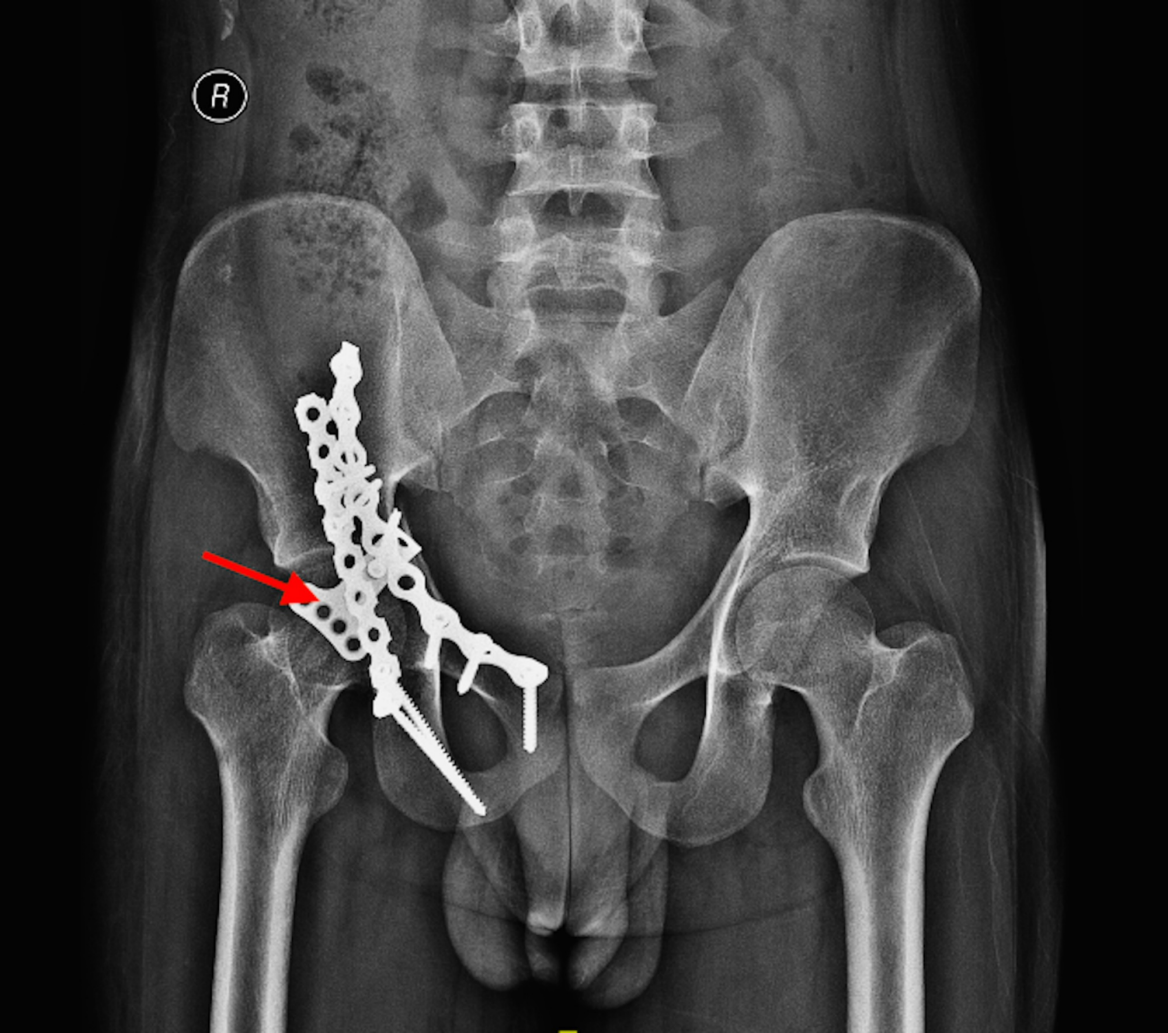
X-ray pelvis shows the right acetabulum fixed internally with plates and screws (marked by arrow)

A 20-year-old male met with a road traffic accident when he was hit by a moving car while riding a two wheeler. He sustained blunt trauma to his abdomen and pelvis. After initial resuscitation at an outside hospital, he was found to have right acetabular fracture for which he underwent open reduction and internal fixation. His post-operative period was uneventful. One month after the trauma, he developed abdominal pain and abdominal distension for one day. He also complained of multiple vomiting episodes for the same period. On examination, the patient was conscious, oriented and was afebrile. His pulse rate was 100/min and blood pressure was 110/70 mm Hg. His abdomen was uniformly distended with diffuse tenderness and guarding. Bowel sounds were absent. His chest X-ray revealed free air under diaphragm and X-ray pelvis showed status internal fixation of right acetabulum with plates and screws (Figures 1, 2).

With the clinical diagnosis of bowel perforation with peritonitis, the patient was taken up for emergency laparotomy.

**Figure 3:**
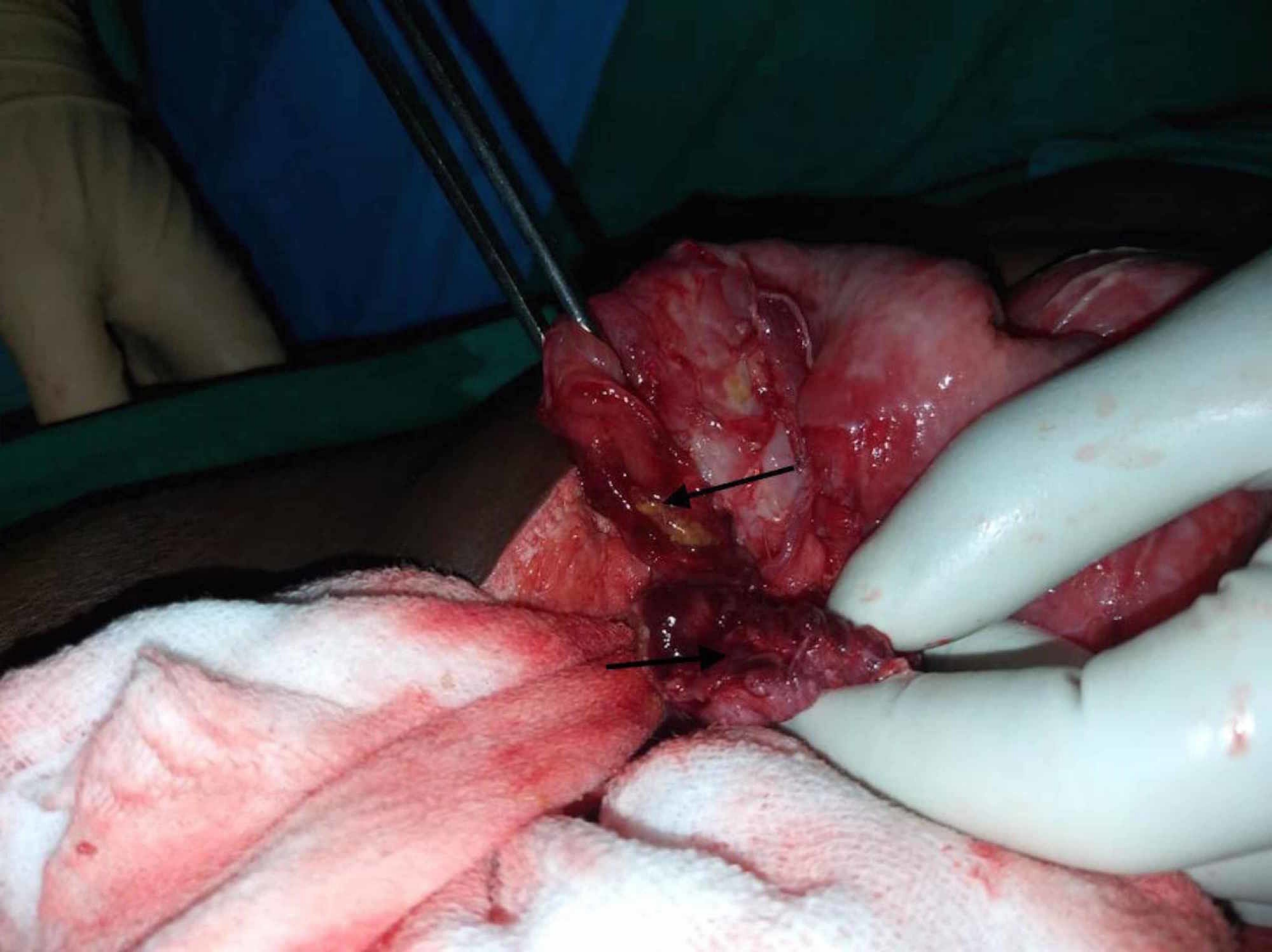
Intra-operative picture showing completely transected ileum (arrows pointing to the lumen)

Intra-operatively, about one-liter purulent contamination associated with pus flakes was noted within the peritoneal cavity. A complete ileal transection was found at about 10 cm proximal to the ileo-cecal junction (Figure 3).

Multiple interbowel adhesions were noted. Other visualized organs were normal. The transected part was brought out as ileal stoma with a distal mucosa fistula. Adhesiolysis was done. Bilateral flank drains were placed. He received intensive care for acidosis and was extubated on post-operative day 2. He was started on oral feeds from post-operative day 5 and was discharged on post-operative day 9. The patient underwent closure of ileostomy after six weeks.

## Discussion

Bowel perforation is reported in less than 1% of patients with blunt abdominal trauma [1]. Isolated small bowel perforation is a rare occurrence in blunt abdominal trauma. The various pathophysiological mechanisms for bowel perforation include crush injury, rapid deceleration and rupture phenomenon [2]. Crushing of the bowel segments is the most common mechanism, and it is usually between the seat belt and the vertebra. Rapid deceleration creates a shearing force at the fixed points of the bowel, namely the duodeno-jejunal flexure and the ileo-cecal junction for the small intestine. The burst phenomenon occurs due to a rapid increase in intraluminal pressure leading to perforation of the bowel wall at the anti-mesenteric border, where the bowel is usually weaker [3]. 

Delayed perforation of the intestine following trauma occurs due to ischemic necrosis, either through direct trauma to the intestinal wall or indirectly by injury to the mesenteric vessels. Mesenteric injuries are the most common cause of delayed intestinal necrosis after blunt abdominal trauma. Mucosal ischemia can lead to ulceration and healing by fibrosis or cicatricial stenosis that can further compromise blood flow to this intestinal segment.

The clinical findings are subtle, and the classical findings of abdominal tenderness, rigidity and decreased bowel sounds are seen in only one-third of the patients. Repeated physical examination is important to pick up the signs early in the course. The clinical evaluation becomes even less effective when there are other associated distracting injuries like head injury causing altered mentation. The only consistent feature is abdominal pain without any significant clinical findings. Therefore, clinical examination should always be combined with radiological investigations to aid in early diagnosis and to prevent unnecessary delays [4].

Serial abdominal X-ray and/or abdominal computed tomography (CT) scan with contrast can aid in diagnosis. An X-ray can show air under the diaphragm in case of bowel perforation. Focused assessment with sonography in trauma (FAST) is a rapid and sensitive method for detection of free intraperitoneal fluid, but it is less accurate in detecting mesenteric ischemia. Findings such as portomesenteric vein gas and intramural gas in the small bowel in the CT scan are suggestive of severe mesenteric ischemia. A contrast CT scan is the imaging modality of choice in stable blunt abdominal trauma patients who continue to have abdominal pain, in spite of its significant false-negative rate [5]. A repeat CT scan can be valuable in patients who develop signs during the observation period.

Laparoscopy can be used as a diagnostic as well as a therapeutic modality in these cases, and they are shown to prevent non-therapeutic laparotomy in up to 40% of the cases [6]. Diagnostic abdominal paracentesis and peritoneal lavage are valuable and effective in detecting hemorrhage after blunt abdominal trauma but are highly non-specific.

Stable patients without clinical signs of peritonitis are managed conservatively. However, the potential for delayed small bowel perforation should be borne in mind and patients warned about the symptoms and counseled regarding the same. Unstable patients and those with evidence of bowel perforation should undergo a laparotomy. Intra-abdominal contamination is usually present and a definitive repair or an anastomosis is likely to break down. There is a threefold increase in morbidity and mortality due to the delayed presentation. There is an increased incidence of postoperative complications like wound infection, wound dehiscence, intra-abdominal abscess, acute respiratory distress syndrome and sepsis adding to the morbidity [7].

## Conclusions

The rarity of isolated small bowel perforation, the paucity of clinical findings on initial admission and limitations of current diagnostic investigations lead to a delay in the definitive treatment of these patients. A detailed history including the mechanism of injury, close observation with repeated physical examinations, thorough investigations with CT and/or FAST, repeated if necessary, and awareness of the possibility of small bowel perforation are cornerstones to successful management in patients with blunt trauma of abdomen.
